# Glycemic Variability in Subjects with Diabetes and Hypogonadism during Testosterone Replacement Treatment: A Pilot Study

**DOI:** 10.3390/jcm11185333

**Published:** 2022-09-11

**Authors:** Giuseppe Defeudis, Ernesto Maddaloni, Giovanni Rossini, Alfonso Maria Di Tommaso, Rossella Mazzilli, Paolo Di Palma, Paolo Pozzilli, Nicola Napoli

**Affiliations:** 1 Unit of Endocrinology and Diabetes, Department of Medicine, University Campus Bio-Medico di Roma, 00128 Rome, Italy; 2 Department of Experimental Medicine, Sapienza University of Rome, 00185 Rome, Italy; 3 Endocrinology Unit, Department of Clinical and Molecular Medicine, Sapienza University of Rome, Sant’Andrea Hospital, 00189 Rome, Italy; 4 Unit of Urology, Hospital of Frosinone, ASL Frosinone, 03100 Frosinone, Italy

**Keywords:** diabetes, hypogonadism, testosterone replacement treatment, glycemic variability

## Abstract

Background: This is a proof of concept, as a pilot study, with the aim to evaluate continuous glucose monitoring metrics (CGM) in subjects with type 2 diabetes (T2DM), treated with nutritional therapy and metformin, before and after testosterone replacement therapy (TRT). Methods: In this longitudinal observational study, subjects affected by T2DM and starting TRT for documented ED and hypogonadism were enrolled. All subjects mounted a CGM system during the v0 visit, one week before the beginning of the TRT (week−1), during v2, four weeks after the start of TRT (week 4), and v4 (week 12). CGM was worn for about 144 h after each visit. Results: A total of seven patients, referring to our clinic for erectile dysfunction (ED), were studied (aged 63.3 ± 2.3 years). Mean (± standard deviation) total testosterone level was 2.3 ± 0.6 ng/mL at baseline. After TRT, total testosterone level was 4.6 ± 3.04 ng/mL at week 4 and 3.93 ± 4.67 ng/mL at week 12. No significant differences were observed in TIR, TAR, TBR, estimated HbA1c, AUC below, and AUC above limit during the intervention period. Conclusions: This is the first study evaluating the effects of TRT on daily glucose excursions in subjects with T2DM and hypogonadism. Though we did not find any significant difference in key CGM metrics during the 12 weeks of TRT, this study confirms the glycometabolic safety of the TRT even on the most novel standardized glycemic targets.

## 1. Introduction

Diabetes mellitus (DM) is a major global health problem, with DM-related complications being a main cause of increased morbidity and mortality. Among these, erectile dysfunction (ED), with a prevalence which is approximately 3.5-fold higher than in men without DM, and male hypogonadism are increasingly recognized as invalidating chronic diabetic complications [1,2,3]. Hypogonadism also affect up to 35–40% of male subjects with DM, mainly type 2 (T2) DM [4,5,6]; the heterogeneity of the results depend on cut offs used to define hypogonadism and the study design [7]. 

Age, insulin resistance, poor glycemic control, comorbidities, such as obesity and OSAS, and other endocrine dysfunctions [8] are recognized risk factors for diabetic ED, and restoring these conditions may benefit the patient by increasing testosterone levels, even without testosterone replacement therapy (TRT) [9]. Furthermore, in patients with diabetes and hypogonadism, TRT can improve sexual activity, as well as quality of life (QoL), insulin resistance, lipid profile, body pressure, and body composition [10,11,12,13]. In addition, a recent meta-analysis demonstrated that TRT do not increase the risk of cardiovascular diseases [14], particularly if the comorbidities are well controlled [15]. Anyway, a lot of studies confirmed the role of hypogonadism in considering itself as a cardiovascular risk factor [16]. 

Although some studies suggested a potential effect of TRT directly on glucose metabolism and glycemic control [17,18,19], evidence in this regard is still limited. In particular, there is a lack of data about the effect of TRT on glycemic variability, which has acquired increased importance as a marker of glycemic control [20]. Conversely, one cross-sectional study evaluating the correlation between endogenous testosterone levels and glycemic variability, assessed by continuous glucose monitoring (CGM) in patients with T2DM, showed that high circulating total testosterone levels were positively correlated with increased glycemic variability, considering these increased levels a potential predictor of glycemic variability [21]; furthermore, these results suggested the potential to evaluate if the safety of TRT, in terms of glycemic variability, could preserved. Anyway, no studies are available that are evaluating the direct effects of TRT on glycemic variability. 

The present pilot study is a proof of concept with the aim to evaluate the short-term effect of TRT on glycemic variability, assessed by CGM, in subjects with T2DM.

## 2. Materials and Methods

### 2.1. Study Design and Population

This is a longitudinal observational study conducted at the Diabetes outpatient clinics of the Campus Bio-Medico University of Rome, Italy. 

In this study, we enrolled, in six months, consecutive patients affected by T2DM and starting TRT for documented ED and hypogonadism.

Exclusion criteria were: (a) type 1 DM; (b) any antidiabetic drug therapy other than metformin; (c) any contraindications to TRT among history of prostate or breast cancer, hematocrit >52%, PSA >4 ng/mL, uncontrolled OSAS and heart failure (NYHA class III/IV), severe lower urinary tract symptoms (AUA/IPSS score > 19), or a desire to preserve fertility; (d) patients using systemic corticosteroids or any drugs that may influence testosterone level in the three months before enrollment.

ED was diagnosed using a validated questionnaire IIEF-5 (International Index of Erectile Function-5) [22]. In case of diagnosis of ED (total score <22), patients were screened for hypogonadism, according to the criteria established by the Endocrine Society guidelines [23]. In case of a double check of total testosterone level (repeated one month after the first evaluation) <2.6 ng/mL, the diagnosis of hypogonadism was confirmed, and the inclusion and exclusion criteria useful to a final enrollment of the patients in the study were evaluated. Each subject signed an informed consent to participate in the study and to use the CGM. 

For each patient, the following baseline parameters were collected: age, weight, height, BMI, systolic and diastolic blood pressure, HbA1c, lipid profile (total cholesterol, HDL cholesterol, triglycerides, LDL cholesterol), GOT, GPT, creatinine, total PSA, total testosterone, complete blood count, presence and extent of microalbuminuria, presence of diabetic retinopathy, presence of cardiovascular diseases and other comorbidities, drug therapy, IIEF-5 and IPSS score.

**Figure 1:**
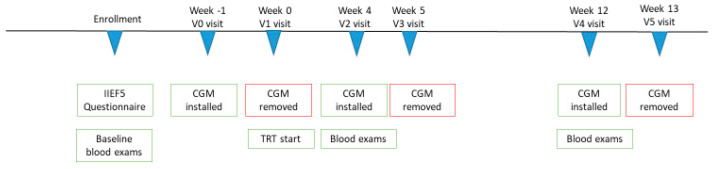
Study design. CGM Continuous Glucose Monitoring; IIEF5: international index erectile Function 5. TRT: Testosterone Replacement Therapy.

All enrolled subjects, during the v0 visit, one week before the beginning of the TRT (week−1), mounted the CGM (Medtronic iPro 2^®^) for about 144 h (6 days) (Figure 1). 

During v1 (week 0), the CGM was disassembled, the recordings were downloaded, and the patient started TRT gel 2, with a standardized dosage of six puffs per day (as indicated in the technical indications of the drug). Each puff contains 10 mg of testosterone.

During v2, four weeks after the start of TRT (week 4), the patient came to the center with the blood tests requested during v1, and the CGM was mounted for additional 144 h. 

During v3 (week 5), the CGM was disassembled, and the recorded data were downloaded. 

During v4 (week 12), the exams required during v2 were evaluated, and the CGM was reassembled for an additional 144 h. 

During v5 (week 13, end of study) the CGM was disassembled, and the recorded data were finally downloaded.

The study was conducted in accordance with the Declaration of Helsinki and approved by the Ethics Committee ASL Roma 2, study n. 85.21.

### 2.2. Assessments

All enrolled participants were equipped with a CGM sensor for 6 days. The CGM system used included a digital recorder, Medtronic iPro 2, and a glucose sensor, Enlite^®^ sensor, MMT-7008. Reports were generated with the CareLink iPro^®^ Therapy Management Software for Diabetes (CareLink iPro, MMT-7340). The following parameters were analyzed: time in range (TIR between 70 and 140 mg/dL), time above range (TAR >140  mg/dL), time below range (TBR <70  mg/dL), high excursion, low excursion, estimated HbA1c, area under the curve (AUC) for blood glucose values above and below the target, and average blood glucose at 3 h intervals.

We evaluated the following parameters extrapolated from the CGM: the average of all glycemic values in 6 days per single patient; average pre-breakfast area under the curve (one hour before breakfast); average area under the post-morning curve (60 min after breakfast); average area under the post afternoon curve (60 min after lunch); average area under the post-evening curve (60 min after dinner). 

### 2.3. Statistical Analysis

Descriptive statistics are presented, for categorical variables, as numbers with proportions, as well as for continuous mean ± standard deviation (SD). Friedman test was used to assess changes of continuous variables over time. A *p* value < 0.05 was considered as statistically significant. All statistical analyses and graphical representations were performed using GraphPad Prism 9.1.

## 3. Results

Eight men were enrolled in the study. One patient was excluded because of incomplete CGM data. Therefore, a total of seven patients aged between 60 and 65 (62.3 ± 2.3 years) were included in the final analyses. All patients were overweight/obese (mean BMI 29.9 ± 2.4 kg/m^2^). Baseline characteristics are described in Table 1.

All patients were referred for ED, and the IIEF-5 value was 10.0 ± 3.3 at baseline and 11.7 ± 4.6 after 3 months of TRT (*p* = 0.6).

Total testosterone levels were 2.1 ± 0.4 ng/mL for the baseline blood works. A total of six puffs daily of testosterone gel was administered.

Medium testosterone levels were 4.6 ± 3.0 ng/mL at week 4 and 3.9 ± 4.7 ng/mL at week 12. None of the subjects developed erythrocytosis, as well as alteration in blood count, or had pathological PSA increase (Table 2). 

**Figure 2:**
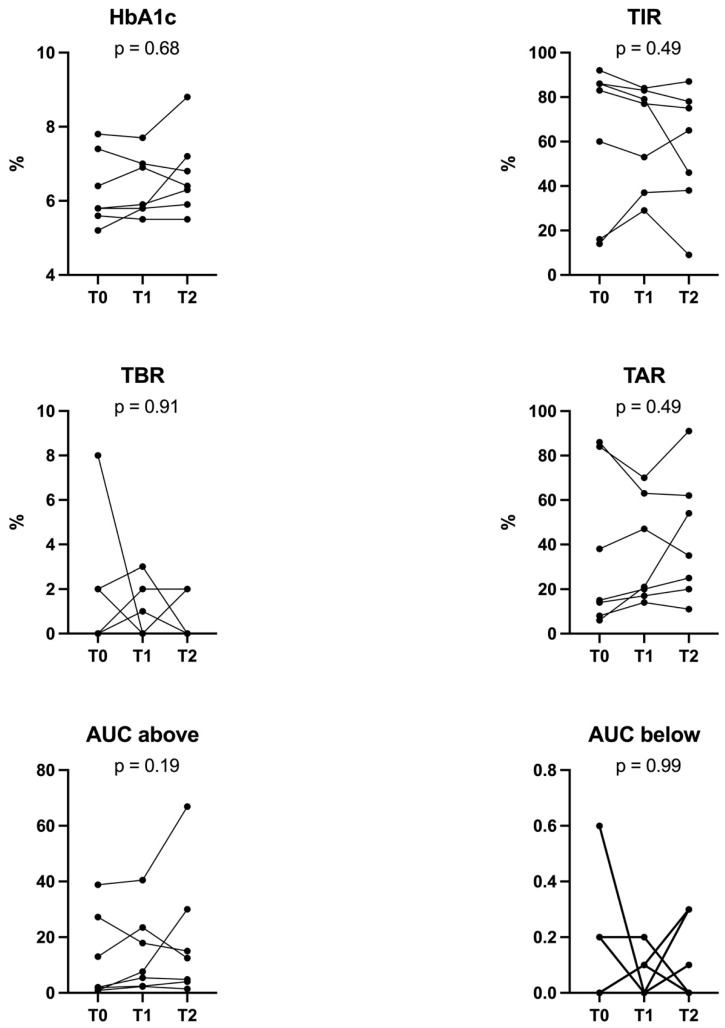
Illustration of Time in range (TIR), time above range (TAR), time below range (TBR), estimated HbA1c, AUC above, and AUC below limit at baseline time and after 4 and 12 weeks of testosterone replacement treatment.

There were no significant changes in TIR, TAR, TBR, HbA1c, AUC below, and AUC above limit during the intervention period (Figure 2, Table 2).

## 4. Discussions

This pilot study assessed the impact of TRT on glycemic variability in men affected by DM and hypogonadism. To date, this evaluation is not yet estimated; anyway, there are many studies that are prsovided to evaluate the role of glycometabolic parameters in these populations, giving the basis to explore the effect of TRT on glycemic variability to literature. In this direction, the TIMES2 study has found that the treatment with 2% testosterone gel promotes a significant reduction in insulin resistance, evaluated with the HOMA-IR, after 12 months of treatment [18]. Furthermore, Shigehara et al. [19] found a significant improvement in HbA1c values, fat percentage, and erectile function, together with a decrease in cardiovascular risk, in patients with diabetes after one year of intramuscular testosterone enanthate (250 mg) injections every four weeks [19]. Interestingly, Yassin et al. have observed that TRT, with testosterone undecanoate injections for 8–11 years, prevents the progression of DM in patients with T2DM, even restoring the euglycemic state [24]. In addition, a long-term real-world evidence study showed that TRT with undecanoate injections promoted the remission of DM in one-third of the patients and the improvement of glycemic control in the remaining subjects [25]. Recently, a randomized, double-blind, placebo-controlled (RTC) study (T4DM trial) highlighted that patients treated with testosterone undecanoate injections showed a significant reduction in the risk T2DM, with a higher proportion of normal fasting glucose and glucose at the after 2 h levels [26].

Ding et al. [21] demonstrated that baseline testosterone levels correlated positively with glycemic variability, expressed as standard deviation of mean blood glucose (SDBG), in Asian men with DM; in particular, a total testosterone level above 14.76 mmol/L (4.21 ng/mL) was a predictor of glycemic variability, according to ROC curves. 

According to the findings of Ding et al., we could speculate that increasing testosterone levels in hypogonadal men with DM could have led to an increase in glycemic variability after TRT. Conversely, our study showed no significant deterioration of any CGM parameters. Anyway, the exposure to TRT differs among the different replacement preparations and regimens [27]; this speculation could result in different effects on glycemic variability. The testosterone preparation we used in our study was a 2% testosterone gel applied daily, in the morning, on the skin of the abdomen; this application caused a peak of testosterone levels between 2 and 4 h after administration and maintained testosterone levels above baseline for approximately 12 h [28]. According to this, we expected to see the biggest impact on glycemic variability in the early hours after testosterone gel administration, but the average glucose levels, calculated by the CGM at 3 h intervals, were not significantly different from pretreatment.

The absence of effects of TRT on glycemic variability should also be interpreted on the basis of the good mean glycemic control of the population enrolled. In this regard, a recent meta-analysis showed that HbA1c improvements during TRT are higher in subjects with baseline HbA1c > 8% [29]. In contrast, the population enrolled in our study had a mean HbA1c of 7.2% (SD +/− 1.8). Furthermore, to isolate the effects of TRT on glycemic variability, we included only people on diet and/or metformin monotherapy, which is associated with lower glycemic variability compared to other antidiabetic drugs, such as sulfonylureas or insulin [30]. In addition, we could speculate that TRT could have a higher impact on glycemic variability in subjects with a baseline worse glycemic control and on different antidiabetic drugs. On the other hand, an inverse correlation between testosterone and insulin levels, outlining a positive relationship between testosterone and insulin sensitivity has been described [31]; anyway, the mechanisms are still unclear.

Finally, the increase in total testosterone after 12 weeks appeared rather moderate, and this may have influenced the outcome; this could be due to the phenotypic features of our population, characterized by overweight/obesity and mainly hypogonadotropic hypogonadism.

The strengths of this study are the longitudinal design, the standardized cut off used to define HG, and the confirmation of biochemical hypogonadism, as well as the evaluation of clinical symptoms using a standardized questionnaire.

The main limitations of this study were: (1) the small number of participants; (2) the absence of a control group; (3) the short evaluation period; (4) no data about the adherence of the subjects to TRT. In this regard, anyway, all of these limitations are inherent in a pilot study that, for the first time, explores glycemic variability in these population. Furthermore, we cannot exclude that a different testosterone formulation could have a different impact on glycemic control and variability.

Future research directions may focus on an evaluation of an alternative of use of TRT in subjects with diabetes, even defining different daytime to take this therapy, depending by glycemic values of the subjects, leading to a “tailor made” treatment. 

## 5. Conclusions

In this first proof-of-concept study evaluating the effects of 12 weeks TRT on CGM metrics, in subjects with T2DM and hypogonadism, we did not find any significant difference in the most novel standardized glycemic targets, confirming the glycometabolic safety of TRT. Further larger studies and RCT should be conducted to confirm this observation. 

## Figures and Tables

**Table 1:** Baseline characteristics of the population.

Age (years; mean ± SD, range)	62.3 ± 2.3 (60–65)
Smoking habits (yes; n., %)	2/7 (28.6%)
Family history of DM (n., %)	7/7 (100%)
Diabetes duration (years; mean ± SD, range)	8.4 ± 2.2 (4–12)
Total Cholesterol (mg/dl; mean ± SD, range)	152.6 ± 32.7 (124.0–203.0)
Triglycerides (mg/dl; mean ± SD, range)	114.0 ± 26.0 (84.0–143.0)
HDL Cholesterol (mg/dL; mean ± SD, range)	46.8 ± 16.4 (33.0–70.0)
Total Testosterone (ng/mL; mean ± SD, range)	2.1 ± 0.4 (1.7–2.5)
LH (mIU/mL; mean ± SD, range)	5.9 ± 4.7 (2.0–15.9)
FSH (mIU/mL; mean ± SD, range)	11.9 ± 10.6 (2.6–31.3)
Prolactin (ng/mL; mean ± SD, range)	4.5 ± 1.2 (3.8–5.5)
Total PSA (ng/mL; mean ± SD, range)	1.1 ± 0.7 (0.3–2.1)
SHBG (nmol/L; mean ± SD, range)	28.7 ± 8.3 8 (19.1–33.7)
Hematocrit (%; mean ± SD, range)	44.2 ± 2.9 (40.2–49.0)
Hb (ng/dL; mean ± SD, range)	15.1 ± 1.1 (13.6–16.7)
BMI (kg/m^2^; mean ± SD, range)	29.9 ± 2.4 (26.4–32.8)

LH: luteinizing hormone; FSH: Follicle-stimulating hormone; SHBG: sex hormone binding globulin; PSA: prostate-specific antigen; Hb: hemoglobin.

**Table 2:** Time in range (TIR), time above range (TAR), time below range (TBR), estimated HbA1c, AUC above and AUC below limit, total testosterone, total prostate-specific antigen (PSA), and hematocrit at baseline time and after 4 and weeks of testosterone replacement treatment.

	Baseline	After 4 Weeks	After 12 Weeks
TIR (%; mean ± SD)	62.4 ± 34.0	63.1 ± 23.2	56.9 ± 27.4
TAR (%; mean ± SD)	35.9 ± 35.2	36.0 ± 23.6	42.6 ± 28.1
TBR (%; mean ± SD)	1.7 ± 2.9	0.9 ± 1.2	0.6 ± 1.0
HbA1c (%; mean ± SD)	6.3 ± 0.9	6.3 ± 0.8	6.7 ± 1.1
AUC above limit (mean ± SD)	12.1 ± 15.3	14.2 ± 14.1	19.2 ± 23.1
AUC below limit (mean ± SD)	0.1 ± 0.2	0.1 ± 0.1	0.1 ± 0.1
Total Testosterone (ng/mL; mean ± SD)	2.1 ± 0.4	4.6 ± 3.0	3.9 ± 4.7
Total PSA (ng/mL; mean ± SD)	1.1 ± 0.7	1.3 ± 0.7	1.2 ± 0.8
Hematocrit (%; mean ± SD)	44.2 ± 2.9	44.7 ± 2.6	46.9 ± 4.9

## Data Availability

Not applicable.
